# In the September 2015 issue

**DOI:** 10.1590/1980-57642015DN93000001

**Published:** 2015

**Authors:** Ricardo Nitrini

**Affiliations:** Editor-in-Chief

In this issue we are publishing two reviews, eleven original papers, one history note and one case report.

**Wajman et al.** analyzed the influence of cultural phenomena on behavior and on cognition and functional realms. The authors concluded that continual research in this area is essential to understand differences in performance on neuropsychological tests among culturally and linguistically different populations.

**Takada** reviewed the genetics of monogenic frontotemporal dementia, an area where several novel discoveries in the last decade have furthered our knowledge and may have an impact on the future diagnosis and treatment of these conditions.

**Tiel et al.** performed a systematic review on the neuropsychiatric symptoms of vascular cognitive impairment and included 13 papers in their analysis. The authors found that subcortical and cortical-subcortical vascular dementia were described predominantly with apathy and depression, whereas neuropsychiatric symptoms were less frequent in vascular cognitive impairment without dementia.

**Damin et al.** designed a 22-item questionnaire to identify individuals with normal cognition, mild cognitive impairment (MCI) and dementia in Brazil. The Questionnaire of Cognitive Change was able to classify individuals into these three groups with high accuracy, even after reducing the number of questions to eight.

**Jacinto et al.** carried out a transcultural adaptation of an instrument developed in the United Kingdom for assessing the knowledge and attitudes of physicians towards dementia. After adaptation to the Brazilian context, the instrument was considered adequate for future validation and use in Brazil.

**Ramanan et al.** investigated category and phonemic verbal fluency tasks in the discrimination of AD from MCI and bvFTD. The numbers of responses and switches in category fluency were able to differentiate AD from MCI in most cases, but verbal fluency tests proved insufficient to discriminate AD from bvFTD.

**De Paula et al.** used modified versions of the verbal fluency tests to evaluate specific aspects of the executive functions. The authors found that a modified version in which the participant had to give the name of an animal followed by a fruit (as word pairs|) was a more specific measure of cognitive flexibility than other verbal fluency tests.

**Serrao et al.** used a Lexical Decision Test in three groups of elderly: healthy individuals, patients with MCI and with AD. Although there were differences in the cognitive tests, the Lexical Decision Test did not differ among the three groups, showing that this test may be used for measuring premorbid intelligence quotient (IQ)

**Sposito et al.** evaluated a large cohort of Brazilian elderly to investigate the relationship between engagement in advanced activities of daily living and performance on the domains of the Mini-Mental State Examination. The authors found an association of engagement in advanced intellectual and social activities with better cognitive performance.

**Silveira and Mansur** analyzed the discourses of normal and aphasic subjects when narrating three selected fairy tales. The number of propositions was lower in aphasic individuals and the three stories were able to differentiate between control and aphasic individuals based on the macrostructure of the discourse.

**Muller et al.** conducted a systematic review on executive functions as a potential neurocognitive endophenotype in anxiety disorder diagnosis using several data bases. Executive dysfunctions were considered a neurocognitive endophenotype in obsessive-compulsive disorder. The authors concluded that further studies on executive functions in other anxiety disorders are needed.

**Artigas et al.** presented data showing that attending a support group for Parkinson disease patients was associated with better quality of life scores and fewer symptoms of depression, anxiety and social phobia when compared to Parkinson disease patients who did not attend a support group.

**Kessels et al.** investigated verbal and visuospatial working memory in mild cognitive impairment (MCI) and in Alzheimer’s disease (AD). This study showed that working memory deficits are present in both MCI and AD, but that impairments are not always evident on all working memory tests.

**Engelhardt and Grinberg** revisited the papers published by Alois Alzheimer on vascular brain diseases to find that he had identified and described three groups of vascular brain diseases that are the main forms we currently recognize as responsible for Vascular Cognitive Impairment.

**Simabukuro et al.** reported a case with a long course of psychiatric disease diagnosed as Bipolar Affective Disorder that evolved to encephalitis caused by antibodies against NMDA receptor. The possibility of a relapsing autoimmune disorder or two conditions was discussed.

**Ricardo Nitrini** Editor-in-Chie

